# Smoking is not associated with higher prevalence of JC virus in MS patients

**DOI:** 10.1007/s10096-018-3204-z

**Published:** 2018-02-08

**Authors:** Michael Auer, Gabriel Bsteh, Harald Hegen, Franziska Di Pauli, Sebastian Wurth, Thomas Berger, Florian Deisenhammer

**Affiliations:** Department of Neurology, Medical University of Innsbruck, Innsbruck, Austria

**Keywords:** JCV, Multiple sclerosis, Natalizumab, Progressive multifocal leukoencephalopathy, Smoking

## Abstract

John Cunningham virus (JCV) causes rare, but potentially life-threatening progressive multifocal leukoencephalopathy (PML) in natalizumab-treated multiple sclerosis (MS) patients. Beside JCV index, there is currently no other factor for further risk stratification. Because smoking was reported as potential risk factor for several viral and bacterial infections, we aimed to investigate whether smoking could increase the risk for JCV infection in MS patients. We screened our database of the MS Clinic of the Department of Neurology, Medical University of Innsbruck, Austria, for patients with known smoking status and test result for anti-JCV antibody index as determined by two-step ELISA at Unilabs, Copenhagen, Denmark. In a representative cohort of 200 MS patients with a rate of 36% current smokers plus 6% former smokers, we were not able to detect any association between smoking and JCV status. Furthermore, there was no association between smoking status and anti-JCV antibody index. Smoking does not seem to be a risk factor for JCV infection in MS patients and, therefore, does not represent a suitable marker for PML-risk stratification under treatment with natalizumab.

## Introduction

Progressive multifocal leukoencephalopathy (PML) is a rare, but potentially life-threatening adverse event during treatment with natalizumab (Tysabri®, Biogen Idec) in MS patients [1]. It is caused by reactivation of John Cunningham virus (JCV). Measuring of anti-JCV antibodies in patients’ serum has become an important biomarker for PML risk stratification and, thus, for treatment decision making as well as for safety management during natalizumab therapy. Recent studies showed JCV prevalence rates around 55–60% in adult MS patients [2–5], of whom only a few eventually develop PML. This underscores the urgent need to further stratify anti-JCV antibody positive patients, i.e., to detect possible risk factors for JCV infection and reactivation. So far, the only confirmed marker which allows narrowing the high PML-risk group within JCV positive patients is JCV index [6].

Up to date, there is no current knowledge about possible risk factors for JCV infection. The route of transmission of JCV has not been discovered so far, whereby environmental risk factors have been discussed as well as gastrointestinal and respiratory route [7–11]. There is some evidence that tobacco smoking may be a risk factor for different viral infections [12], although there is no study investigating possible coincidence of JCV infection and smoking so far. Since smoking is an easily assessable risk factor, we aimed to investigate a possible coincidence of smoking and JCV infection in MS patients in order to detect a potential influence of respiratory tract in transmission of JCV.

## Methods

We obtained our patient data retrospectively from the specific database used in the MS Clinic of the Department of Neurology at the Medical University of Innsbruck, Austria. All demographic and clinical data including smoking habits as well as diagnostic and treatment data have been entered in this database for more than 10 years. We included patients of whom smoking status, gathered during routine visits, was available for different time points prior, during, and after onset of MS, and of whom at least one JCV test was available. Additionally, all JCV stratify-test results including JCV index where collected in order to allow a follow-up of JCV status during treatment.

A general vote of ethics committee for use of anonymized retrospective data was obtained. Anti-JCV antibodies (IgG subclass) were routinely tested by a two-step ELISA (STRATIFY JCV DxSelect™ [13]) at Unilabs, Copenhagen, Denmark. We obtained qualitative (JCV negative/positive) as well as quantitative results, the latter expressed by an OD (optical density) which allows quantifying presence of anti-JCV antibodies and stratifying patients into negative, low positive ( <1.5) and high positive (> 1.5) for anti-JCV antibodies.

For statistical analysis, Graph Pad Prism 6 (Graphpad Software Inc., La Jolla, CA, USA) was used. Distribution of data was tested using D’Agostino-Pearson normality test. According to distribution and category, data are shown as median and range or mean ± standard deviation as appropriate. Smoking habits and JCV status were correlated using chi-square-test, group comparison of JCV index between smokers and non-smokers was performed by Mann-Whitney-test. *P* values of < 0.05 were considered statistically significant.

## Results

**Fig. 1:**
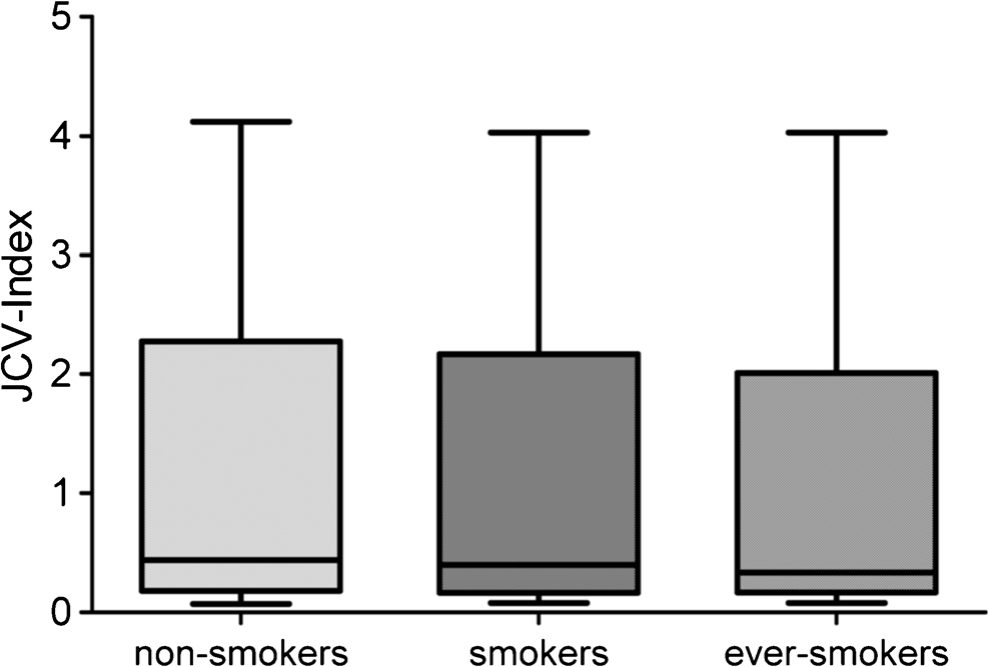
JCV indices (expressed by OD of JCV-ELISA) in non-smokers, current smokers and ever-smokers. Ever-smokers include all current smokers and patients who had smoked at any time and stopped smoking before onset of MS. There was no difference of JCV index between the groups (*p* > 0.05)

We included those patients in our analyses of whom smoking status before, during, and after onset of MS as well as at least one JCV test were available which resulted in a study cohort of *n* = 200. The cohort included 153 (76.5%) female and 47 (23.5%) male patients. Median age at time of first JCV testing was 35 (14–54) years. Sixty-four (32%) patients were persistently JCV negative, and 114 (57%) were persistently positive. Six patients (3%) switched from negative to positive status during follow-up testing and 16 (8%) patients converted/reconverted at least once between negative and positive around the cut-off index. Seventy-two patients (36%) were smokers, 12 (6%) stopped smoking before onset of MS and, consecutively, before JCV testing, while 116 (58%) patients where ever non-smokers. Table 1 shows results of association of smoking habits and JCV status (negative/positive), whereby there was no difference of JCV prevalence between smokers and non-smokers. Switchers of JCV status were analyzed separately. Among the six patients converting from negative to positive JCV status, five were smokers, whereas in the group of patients fluctuating around the cut-point between negative and positive, six patients smoked and ten were non-smokers. No difference of anti-JCV antibody index was found between smokers and non-smokers (Fig. 1). Median JCV index was 0.436 (0.070–4.120) for non-smokers and 0.398 (0.080–4.030) for smokers with a *p* value of 0.298.

**Table 1:** Association of smoking-habits with JCV status

	JCV negative	JCV positive	total
A			
Non-smokers	38	67	105
Ever-smokers	26	47	73
Total	64	104	178
B			
Non-smokers (with former smokers)	43	74	117
Current smokers	21	40	61
Total	64	104	178

The table shows number of patients categorized by smoking habits and JCV status, whereby only permanently JCV negative or positive patients are included. Twenty-two patients of whom six real seroconverters (from negative to high-positive) and 16 patients with borderline JCV results, i.e., fluctuating between negative and borderline-positive status, were excluded. Table 1 A includes 12 former smokers to the group of ever-smokers, Table 1 B includes former smokers to the group of current non-smokers. *P* values are 0.937 for Table 1 A and 0.874 for Table 1 B

When including patients who stopped smoking before JCV testing, median JCV index for ever-smokers was 0.332 (0.080–4.030) with no significant difference as compared to non-smokers (*p* value of 0.474).

## Discussion

We investigated a representative cohort of MS patients for possible coincidence of smoking and JCV infection. Our study cohort was predominantly female according to higher prevalence of MS in women [14]. JCV prevalence at initial testing was 57% which is in agreement with previously published data [2–5]. Thirty-six percent of patients were current smokers, percentage of ever-smokers was 42%. These data coincide approximately with official smoking statistics [15]. A slightly higher prevalence of smokers in MS patients in comparison with normal population is to be assumed due to higher coincidence of psychiatric morbidity in patients with chronic diseases such as MS, depression, anxiety, and addictive behavior [16, 17]. In our study, we were not able to confirm any association of JCV infection and smoking habits, neither with JCV status (negative/positive) nor with JCV index. Transmission of JCV is still poorly understood. Since smoking with all known influences to the respiratory tract seems not to play a role for JCV infection, the theory of viral transmission via the respiratory route cannot be further supported by our study. There have been several attempts to investigate transmission route of JCV. Berger et al. [18] investigated content of JCV-DNA in body fluids such as oropharyngeal fluid, blood, and urine; however, they only detected a considerable amount of copies by polymerase chain reaction (PCR) in urine why they suggested that urine could contribute to transmission of JCV. Other authors suggest a role of respiratory transmission as well due to the presence of virus particles in stromal cells of tonsils and oropharynx [10]. Vanchiere et al. [11] investigated stool specimens, where they were able to detect presence of JCV in the gastrointestinal tract of some subjects (more children than adults) and discussed a potential role of fecal-oral transmission of polyomaviruses. By excretion in urine or stool, polyomaviruses could be released to the environment. Studies detected JCV in about 98% of sewage specimens and confirmed a high stability of the virus outside the human body [7–9]. Due to the ubiquity of JCV and based on sequence analyses of the virus, these authors concluded a potential role of JCV intake via contaminated water or food. Furthermore, there is evidence that JCV infection in children occurs both as vertical transmission from parents to children as well as outside family, indicated by detection of virus DNA-strain-subtypes in urine of children which were partially (in 50%) identical in parents and their children, but diverse in the other half of investigated subjects [19, 20].

Similarly, there is still poor evidence for the role of smoking in triggering or facilitating viral infections. Smoking may have different mechanisms of interference with the immune system and may affect different immunological pathways. A recent review described the current knowledge about interaction of tobacco smoking with different cell types of the innate and adaptive immune system including up- and downregulation of cytokine cascades [21]. Moreover, a decreased level of circulating immunoglobulins and depression of antibody response as well as decreased release of proinflammatory cytokines in smokers has been described [12]. Furthermore, irritation of epithelial structures of the respiratory tract by smoke may lead to adaptive responses of the immune system [22]. Altogether, nicotine and other tobacco components seem to have immunosuppressive effects which may lead to higher susceptibility to bacterial and viral infections. Several studies estimated a higher risk of upper and lower respiratory tract infection in smokers [23, 24]. For influenza, similarly, a higher incidence, and, especially more severe symptoms were found in smokers compared to non-smokers [25, 26]. Also, for HPV (human papilloma virus) and HIV (human immunodeficiency virus), a higher prevalence in smokers was described; however, particularly for these viral infections high-risk behaviors such as sexual practices have to be considered as potential confounders [12]. One study could not detect a relationship between infection with BK-virus, a polyomavirus such as JCV, and smoking as well as other demographic variables [27]. One study investigated a potential oncogenetic role of JCV in lung cancer [28]. To our knowledge, this is the only study investigating the association of JCV and smoking so far, demonstrating a lack thereof (so far there was only one study where this association was investigated as a secondary endpoint of a multivariate analysis). Since we also did not find any relation between smoking and JCV infection in a sufficiently representative cohort, we conclude that smoking habits seem not to contribute additional stratification potential for selection of a high-risk PML group in natalizumab-treated MS patients.

Furthermore, JCV infection seems to occur largely independently from modifiable risk factors.
